# Standards for lithium monitoring. “Are we good at adhering to these standards in Lanarkshire”?

**DOI:** 10.1192/bjo.2021.473

**Published:** 2021-06-18

**Authors:** Saba Ansari, Khalid Nawab, Niall Killeen, Elizabeth Spence, Monika Fotedar

**Affiliations:** NHS Lanarkshire

## Abstract

**Aims:**

The National Institute for Health and Clinical Excellence (NICE) recommend that renal and thyroid function must be checked before lithium is prescribed and for all patients who are prescribed lithium should have their renal and thyroid function checked every six months, and their serum lithium checked every three months. The aim of this audit is to ascertain whether or not routine blood monitoring for bipolar disorder patients, taking lithium is in keeping with the guidelines.

**Background:**

Lithium has been a mainstay in the management of bipolar disorder since the 1970's; indeed, lithium carbonate was first used in the early 1880's for the treatment of mania. Despite its usefulness however, the drawback of lithium treatment remains its very narrow therapeutic index, toxic side effects and as such its need for close therapeutic monitoring.

**Method:**

A list of patients with a diagnosis of bipolar disorder being treated with lithium was collated from an electronic database of psychiatry patients in Cumbernauld Community and inpatients at Glencairn unit Coathill Hospital and Cleland Hospital. A retrospective analysis using Clinical Portal was conducted looking at blood results; Lithium levels checked 3 times a year and Kidney functions and Thyroid function checked twice a year, over the previous year. Our results were then compared to the NICE Guidelines for lithium monitoring to see if they complied with the expected routine monitoring schedule. We may have missed patients open to Community Psychiatric Nurses (CPN) but not open to Consultant psychiatrists. Other group that might have been missed could be open to General Practitioners but not to secondary care. We attempted to contact them but this was unsuccessful.

**Result:**

Total of 690 patients were studied. Of 690 patients 51 patients had the diagnosis of Bipolar Affective Disorder. 49 percent of them were prescribed Lithium. 48percent had their Lithium bloods checked and 60 percent had their Kidney function and thyroid functions checked according to the guidelines. There were no data available for around 7 percent of patients but their Lithium levels were indicated only in Clinical notes.

**Conclusion:**

This audit has demonstrated that Lithium monitoring falls short of conforming to accepted standards. Data obtained by this audit have prompted an electronic alert system for patients on Lithium endorsing primary care, mental health and laboratory staff to work together to ensure supporting recommended Lithium monitoring.

